# Nursing Educators’ Perceptions of AI in Research: Risks and Benefits

**DOI:** 10.1155/nrp/2058036

**Published:** 2026-01-07

**Authors:** Majd T. Mrayyan, Ahmad K. Al-Omari

**Affiliations:** ^1^ Department of Community and Mental Health Nursing, Faculty of Nursing, The Hashemite University, P.O. Box 330127, Zarqa 13133, Jordan, hu.edu.jo; ^2^ Directorate of Nursing, Jordanian Royal Medical Services, Amman, Jordan, jrms.mil.jo

**Keywords:** artificial intelligence, attitudes, educators, ethics, nursing, research, technology acceptability

## Abstract

**Background:**

The application of AI in nursing research is increasing, enhancing objectivity and productivity while raising concerns about liability and scientific integrity.

**Aim:**

To explore predictors and differences in nursing educators’ perceptions of the risks and benefits of using AI in nursing research.

**Methods:**

A cross‐sectional study surveyed 311 nursing educators from various universities.

**Results:**

27% used ChatGPT, and 61.58% were from governmental universities. High perceived risks included liability (*M* = 3.78, *SE* = 0.036), unregulated standards (*M* = 3.76, *SE* = 0.035), and communication barriers (*M* = 3.74, *SE* = 0.036). Perceived benefits included reduced costs (*M* = 3.88, *SE = *0.045) and improved outcomes (*M* = 3.81, *SE = *0.045). Predictors included marital status (*B* = 7.67, *p* = 0.001), age (*B* = −14.65, *p* = 0.001), level of education (*B* = 13.80, *p* = 0.001), academic rank (*B* = −1.755, *p* = 0.001), and teaching experience (*B* = 2.793, *p* = 0.001). The model was significant (*F* (df = 7) = 86.82, *p* = 0.001, *R* ^2^ = 0.660) and explained 66.00% of the variance in the mean score of the perceived use of AI in nursing research. There are significant differences in nursing educators’ use of AI in nursing research based on their age (*F*‐test = 27.63, df = 4, *p* = 0.001), academic rank (*F*‐test = 60.79, df = 5, *p* = 0.001), and teaching experience (*F*‐test = 17.02, df = 4, *p* = 0.001).

**Conclusions:**

Educators recognize both risks and benefits of AI in nursing research. Tailored training and institutional support are essential for responsible adoption. Tackling these issues can pave the way for nursing research to flourish in a rapidly changing digital world. Nursing educators need the tools they need to critically interact with AI in research.

**Reporting Method:**

The authors of this manuscript have adhered to the STrengthening the Reporting of OBservational Studies in Epidemiology (STROBE) checklist, which was used to guide the study.

**Patient or Public Contribution:**

There was no patient or public contribution, as the sample included nursing educators from two governmental universities and one private university.

**Summary**

• Nursing educators’ age, academic rank, and teaching experience predicted the use of artificial intelligence (AI) in nursing research.
• These variables cause differences in nursing academics’ perceived use of AI in nursing research.
• While younger and midcareer educators are more eager to embrace AI tools because they believe in their usefulness and find them easy to use, educators in clinical roles or those with more teaching experience tend to be more cautious.

## 1. Introduction

The first mention of AI in the Medline database was in 1985 when Ryan [1] introduced expert systems to provide clinical decision support and when Sitompul and Randhawa [2] introduced nurse scheduling models. Since AI was first introduced, there have been challenges associated with using AI in nursing in general [3], which also applies to nursing research. AI is the general umbrella used to describe the methods developed to train computers to imitate human cognitive functions, such as communication, learning, making decisions, and reasoning [3, 4]. AI has a wide‐reaching impact on many aspects of science, industry, and technology [4]. AI research fields include machine learning (ML), search algorithms, expert systems, natural language processing (NLP), evolution algorithms, deep learning (DL), and knowledge graphs [4]. Advances in AI methods in the last decade are divided into two main categories: predictive AI tools, which analyze patterns in training datasets and make predictions about new data, and generative AI tools, which generate new data based on patterns observed in datasets [5].

AI has drawn significant attention from various sectors and is now a cutting‐edge tool in healthcare [6]. Over the past 10 years, more studies have been conducted on AI‐based health technologies, with applications showing significant promise for enhancing patient care [3, 6, 7]. Despite the increasing use of AI in healthcare, a significant research gap exists regarding the perceptions of nursing educators concerning its implications, underscoring the necessity for studies that investigate their perspectives to facilitate responsible and contextually appropriate integration of AI in nursing research. In a related study, Algunmeeyn and Mrayyan [8] explored nursing students’ perceptions of AI in nursing research, revealing both enthusiasm and caution toward AI integration. Their findings highlighted the educational dimension of AI adoption and the need for curriculum reform to support AI literacy among future nurses.

### 1.1. Theoretical Framework, Purpose, and Significance

This study focused on nursing educators who are researchers in terms of their perceptions of the risks and benefits of using AI in nursing research. This study draws on the technology acceptance model (TAM), which suggests that how useful and easy to find technology can shape their attitudes toward it and their intention to use it [9]. The TAM has been extensively utilized in healthcare and education to understand technology adoption. It highlights two main beliefs—perceived usefulness and perceived ease of use—that shape users’ attitudes and intentions. In this study, TAM helps explain how demographic and professional factors might influence nursing educators’ views on AI and their perceptions of incorporating it into research. In this research, the perceived usefulness is demonstrated through nursing educators’ perspectives on the advantages of AI in nursing research, including improved data analysis, innovation, and enhanced research outcomes. Perceived ease of use relates to their views on the accessibility, comprehensibility, and manageability of AI tools in the realm of nursing research. These two elements influence educators’ attitudes toward AI and affect their willingness to incorporate such technologies into their research methodologies. By investigating the risks (such as ethical issues and data bias) and benefits (like efficiency and innovation), the study applies the constructs of the TAM to examine how these perceptions differ based on demographic and professional variables.

Nursing educators are essential in molding the future generation of nurse researchers, and their viewpoints affect curriculum development, academic discussions, and the institutional embrace of new technologies [10]. As AI transforms healthcare research, it is crucial to comprehend how nursing educators view its incorporation, especially regarding ethical responsibility, pedagogical significance, and research integrity [11, 12]. This study fills a significant void by examining how these educators assess both the perceived risks, such as possible data biases and ethical concerns related to AI use in nursing research, and the perceived benefits, including improved data analysis and innovation in nursing research [13, 14].

As AI in nursing research is promising, many nursing professionals may need to embrace and adapt to AI‐based technologies as the nursing and healthcare landscape continues to evolve. While this study did not directly assess the role of educators in positioning nursing within the digital future, which is the transformation of the nursing profession through the integration of digital technologies, such as AI into everyday practice, education, and research [15], it applies TAM to equip educators to navigate this future, characterized by increasing reliance on AI and data technologies in nursing research [3]. By linking TAM constructs to educators’ perceptions, the study highlights their readiness to engage with AI tools in research. Nursing educators, especially those who are younger or in the middle stages of their careers, are open to using AI. This openness could really shape the future of nursing research. By providing these educators with specialized training and supportive institutional policies, research practices could be enhanced, and the quality of patient care and professional growth could be improved [16].

Thus, the research questions addressed were as follows: (1) What are the risks and benefits of using AI in nursing research, as perceived by nursing educators? (2) What are the associations of the risks and benefits of using AI in nursing research, as perceived by nursing educators? (3) What are the demographic differences in the perceptions of the risks and benefits of using AI in nursing research, as perceived by nursing educators?

Findings from this research will aid in strategic decision‐making, direct professional growth, and facilitate the establishment of AI frameworks that are ethically robust and contextually aligned with the principles of the nursing profession [17]. The findings may also serve to guide the creation of specialized training programs, institutional policies, and ethical standards that respond to the concerns of nursing educators, while also utilizing their perspectives to effectively incorporate AI tools into nursing research.

## 2. Background

As AI becomes more prevalent in healthcare and nursing practice, the literature highlights several gaps concerning nursing educators’ views on its perceived benefits within research environments. One major gap is the absence of contextual and cultural analysis of using AI in nursing research; most investigations are carried out in high‐income or technologically advanced environments, neglecting the influence of cultural, institutional, and resource‐related factors [13].

Nursing educators and nurses are in a unique position to influence the development of AI in nursing research since they are the users of AI‐based technologies and authorities in academic writing and professional care [3]. However, there is a scarcity of studies focused on educators; most research emphasizes clinical applications of AI or its effects on patient care, with little focus on how nursing educators perceive its application in research contexts [11]. Furthermore, nursing educators, researchers, and nurses are commonly ignored when designing AI‐based technologies [3, 15].

We currently live in the AI age, and the widespread use of AI tools in science has led to a phase where we produce more but comprehend and interpret less [5]. There are perceived risks associated with using AI in nursing, including ethical issues, such as data privacy, algorithmic bias, and the potential decline of clinical judgment. Still, few studies systematically examine how educators assess these risks in relation to possible benefits [13].

Despite these risks, AI has lowered costs and improved the effectiveness of healthcare services [3, 6]. AI in nursing can free researchers and nurses from administrative duties, allowing them to focus on essential components of scientific nursing research and care. AI has transformed nursing care procedures, enhanced care delivery, and improved patient outcomes [6, 15].

In conclusion, when properly designed and implemented early, AI‐based technologies in nursing and nursing research should be easy to use and intuitive [3, 4]. It will be more difficult to address these risks if AI tools become deeply ingrained in nursing research, so AI‐nursing scientists need to assess them now while AI applications are still in early development [5].

## 3. Methods

The study aimed to measure the predictors and differences in nursing educators’ perceptions of the risks and benefits of using AI in nursing research.

### 3.1. Research Design

A quantitative cross‐sectional design was applied using an online survey to assess nursing educators’ perceptions of risks and benefits of using AI in nursing research. Without manipulating the variables, a cross‐sectional study allows data to be collected all at once from a variety of different participants [18]. It is advantageous to use this design to answer different kinds of research questions. Moreover, this design makes the preliminary data collection complete quickly and cheaply [18]. Finally, this nonexperimental descriptive design also incorporated predictive modeling and correlation analyses to explore associations among variables. However, this design has many limitations, such as response bias and the lack of causal relationships. Thus, results should be interpreted with caution.

### 3.2. Sample

The investigation centered on nurse educators who are researchers as its general population. The target population was nursing educators working across different universities. The accessible population for the study was nursing educators from the selected universities, using purposive sampling. A group of 311 nursing educators who were working in nursing programs at the university level in different countries participated in this study, representing a nonprobability convenience sample. Although this approach helped in recruiting a large and diverse sample, the use of nonrandom sampling and broad inclusion criteria may have limited the generalizability to internationally nursing educators, and it may have decreased the internal validity of the findings.

The sample recruitment started at two governmental and one private university in Jordan; these were selected based on the first author’s professional collegiality. While this study brought together participants from a variety of countries, the mix of nationalities may have added a layer of complexity that makes it harder to draw clear conclusions. Focusing solely on nursing educators in Jordan could have provided a dataset that is more cohesive and easier to interpret.

The inclusion criteria were set as that participants were included if they integrated the AI in their nursing research within academic programs, including those in clinical roles but doing research, whether they were principal investigators or coauthors, regardless of the number of research articles published or prepared or years of experience; this helped to recruit a larger sample. However, the broad inclusion criteria may have introduced variability in participants’ exposure to AI, potentially affecting the consistency of responses. The exclusion criterion was set as educators who were not engaged in research.

The first researcher announced the study on her social media platforms, allowing educators to decide whether to take part. Screening questions were used to ensure the target population of nursing faculty members was reached. The study focused on AI as the primary dependent variable, along with nine other nursing educators’ characteristics (independent variables) of gender, marital status, age, education level, type of current and graduate institutions, academic rank, and teaching experience. Although the inclusion of demographic data from educators in various countries enriched the dataset, it also introduced contextual diversity that may complicate interpretation. To enhance the predictive modeling and effect size, these variables were entered into the analysis. However, since the majority of participants were from Jordan, the conclusions were cautiously drawn with the Jordanian context in mind.

The sample size was calculated using the formula of *N* = 10 (*k*) + 50, where *k* represents the number of variables. In this study, *k* = 18, including 10 subscales related to AI perceptions and eight demographic variables. Based on this formula, the minimum required sample size was 230. To enhance statistical power and account for potential variability, a total of 311 nursing educators were recruited, exceeding the calculated requirement. For predictive modeling and to decrease Type II errors, the total score of the scale, rather than the total scores of the subscales, was used.

### 3.3. Ethical Considerations and Data Collection Procedures

The study was approved by the university where the first author currently works. It was confirmed by the Institutional Review Board (IRB) on January 15, 2023, with reference number 22/4/2022/2023; it was related to a comprehensive study about digital leadership/nursing informatics and leadership. The survey was online, and participants had to opt in or out. The first researcher maintained a password for the coded responses in Google Drive, and all participant data were collected anonymously; the form was designed not to collect participants’ emails. Confidentiality was maintained by only providing the overall results to nursing administrators at nursing schools of designated universities so that they can design needed interventions.

Data were collected online in February 2024 using Google Forms for a self‐report survey conducted in English. The survey link was shared by the initial researcher on the personal Facebook pages and WhatsApp of the Faculty of Nursing and the researcher’s colleagues. Nursing educators were asked to participate in the survey, which served as a consent form and an invitation to their contacts. After a week, participants were reminded to complete the survey just once, and information was gathered over 15 days.

### 3.4. Instrumentation

Esmaeilzadeh [19] created and validated an instrument that assessed the “Use of AI‐based tools for healthcare purposes.” The scale consists of 10 subscales with a total of 54 items rated on a 5‐point Likert scale ranging from 1 (strongly disagree) to 5 (strongly agree). The current researcher adopted and adapted the instrument to measure the perceived risks and benefits of using AI in nursing research in the present study. So, this study focused on the perceived risks and benefits of using AI in nursing research. Scores above the midpoint of 3 suggest generally favorable attitudes among participants [20]. The instrument was originally developed to measure the use of AI in healthcare [19]. The adaptation involved rephrasing items to reflect nursing research contexts. Content validity was confirmed by expert panel review using Lynn’s method [21]. In this method, a panel of subject matter experts independently rates each item for relevance to the construct being measured, typically using a four‐point ordinal scale. The content validity index (CVI) is then calculated for each item (I‐CVI) and for the overall scale (S‐CVI), with values above 0.78 for I‐CVI and 0.80 for S‐CVI generally considered acceptable. By using this method, the present study ensured that the adapted instrument accurately reflected the unique context and constructs of AI use in nursing research, as confirmed by expert consensus [21].

Acknowledging the specific contextual and professional subtleties inherent to nursing research, the first author adjusted the original items to ensure they were relevant to the domain. This modified version was employed in the current study. To determine content validity and support conceptual consistency with the intended constructs, a panel consisting of three doctoral‐level experts in nursing leadership research assessed the instrument. Each expert evaluated the relevance of the individual items using a four‐point Likert scale, in accordance with Lynn’s [21] standards. There was a satisfactory consensus among the experts, thereby affirming the instrument’s content validity within the realm of nursing research. It is mindful to mention that before any data collection, the study was piloted with 20 nursing educators to check for its applicability in the research context, and no changes were needed.

Perceived performance anxiety (5 items), perceived social biases (5 items), perceived privacy concerns (6 items), perceived mistrust in AI mechanisms (5 items), perceived communication barriers (5 items), perceived unregulated standards (5 items), perceived liability issues (6 items), perceived risks (very low/very high) (5 items), perceived benefits (7 items), and intention to use AI‐based tools (5 items) are among the subscales of the tool that are related to the perceived risks and benefits of using AI in nursing research. “Taken all together: How positive or negative do they feel about the use of AI in nursing research?” is the general question that the first author added to assess the overall perception toward using AI in nursing research, and it was measured as 5 = very positive, 4 = positive, 3 = neutral, 2 = negative, and 1 = very negative. Any score above the midpoint of 3 suggests generally favorable attitudes among participants [20].

Esmaeilzadeh [19] used the standardized factor loading, composite reliability, and the average variance extracted (AVE) to determine the original scale’s convergent validity. All of the AVEs’ square roots demonstrated discriminant validity, which was higher than 0.700 and greater than the correlations between any two constructs. Along with calculating the Cronbach’s alpha for each construct, Esmaeilzadeh [19] also determined the internal consistency of the instrument (perceived benefits = 0.940, perceived risks = 0.900, performance risks = 0.910, perceived social biases = 0.880, perceived privacy concerns = 0.940, perceived mistrust in AI mechanisms = 0.920, perceived communication barriers = 0.930, perceived unregulated standards = 0.940, perceived liability issues = 0.940, and intention to use AI‐based devices = 0.940). In the current study, the overall Cronbach’s alpha was 0.911, and the values for the subscales were perceived benefits = 0.930, perceived risks = 0.845, performance anxiety = 0.896, perceived social biases = 0.870, perceived privacy concerns = 0.927, perceived mistrust in AI mechanisms = 0.909, perceived communication barriers = 0.876, perceived unregulated standards = 0.877, perceived liability issues = 0.912, and intention to use AI‐based devices = 0.966. This result shows that the instrument is consistent internally.

### 3.5. Data Analyses

The data were analyzed using the Statistical Package for the Social Sciences (SPSS) Version 26 [22], accepting a value of 0.05 as statistically significant. The current research treated the perceived risks and benefits of using AI in nursing research, and the final added question assessed the overall perception toward using AI in nursing research as an interval variable. Descriptive statistics, including means, standard deviations, frequencies, percentages, 95% confidence interval (CI) of the mean, standard errors, and the range, were reported according to the types of variables. The nursing educators’ reporting of high mean scores for perceived benefits and intention to use AI in nursing research, alongside notable concerns about perceived risks, reflects an overall positive perception of AI. Scores above the midpoint of 3 suggest generally favorable attitudes among participants [20].

For the predictive model, the total score of the scale, rather than the total scores of the subscales, was used. The generalized linear model (GLM) was utilized to assess whether the sample’s characteristics (independent variables) were related to the perceived risks and benefits of using AI in nursing research (dependent variable) [18]. Depending on the sample’s characteristics, variances in the perceived risks and benefits of using AI in nursing research (continuous variables) were assessed using *T*‐tests (for two groups) or analysis of variance (ANOVA) followed by Scheffe’s post hoc test (for more than two groups) [18].

## 4. Results

### 4.1. Sample’s Characteristics

Out of a possible 505 nursing educators, 311 working in governmental university nursing schools participated, resulting in a response rate of 61.58%. The majority were married females, mostly aged 35–44, and held postgraduate nursing degrees from both international and national governmental universities. Most participants were in the middle stages of their academic careers, with many (*N* = 120, 38.60%) having 10 or more years of teaching experience. Only a small proportion were full professors (*N* = 44, 14.10%) or associate professors (*N* = 27, 8.70%).

Interestingly, some participants reported not having used any AI tools, possibly due to varying interpretations of what constitutes AI use, ranging from writing assistants like ChatGPT to more advanced applications such as predictive modeling. This finding highlights the need for future studies to include clearer screening questions to distinguish between different types and levels of AI usage in nursing research. Among those who had used AI, ChatGPT was the most commonly mentioned tool (*N* = 84, 27.00%) (Table 1).

**Table 1:** Nursing educators’ characteristics (*N* = 311).

Variables	*N*	%
Gender		
Male	117	37.60
Female	194	62.40
Marital status		
Single	94	30.20
Married	182	58.50
Separated or divorced	8	2.60
Widowed	27	8.70
Age		
Less than 25 years	9	2.90
25–34 years	34	10.90
35–44 years	169	54.30
45–54 years	62	19.90
55 or more	37	11.90
Level of education		
Undergraduate degree	124	39.90
Postgraduate degree	187	60.10
Type of current organization where educators worked		
Governmental	311	100.00
Type of current organization where educators graduated from		
Governmental/public/state	311	100.00
Private	—	—
Academic rank		
Full professor	44	14.10
Associate professor	27	8.70
Assistant professor	64	20.60
Teacher	33	10.60
Assistant teacher	—	—
Clinical trainer	87	28.00
Laboratory technician	56	18.00
Teaching experience		
Less than 1 year	15	4.80
1–2 years	51	16.40
3–4	61	19.60
5–9	64	20.60
Ten or more	120	38.60
Country		
Jordan	210	67.50
United States	33	10.70
United Arab Emirates	29	9.30
Palestine	15	4.80
Saudi Arabia	13	4.20
Qatar	11	3.50
Using any AI tool in nursing research		
Yes	130	41.80
No	181	58.20
AI tool used in nursing research		
ChatGPT	84	27.00
POE	12	3.90
Grammarly	8	2.60
Others	13	4.20
None	194	62.30

### 4.2. Perceived Risks and Benefits of Using AI in Nursing Research

For the interval variables measured on a 5‐point Likert scale ranging from 1 (strongly disagree) to 5 (strongly agree), the overall mean score of the scale of perceived risks and benefits of using AI in nursing research, as perceived by nursing educators, was 3.36 (*SE* = 0.020), indicating a positive perception. All values above 3 indicate a positive or high perception; values near 3 are closer to neutral.

The overall mean for perceived risks of using AI in scientific research was high (*M* = 1.64, *SE* = 0.021). This scale was on a two‐point one, and it was a particular question rather than a subscale. These perceived risks were particularly related to the perceived risks of liability issues, unregulated standards, communication barriers, privacy concerns, performance anxiety, social biases, and mistrust in AI mechanisms; the highest mean indicated the highest perceived risks. More detailed means and standard errors are available in the designated table for all variables.

On the other hand, the overall mean for the perceived benefits of using AI in scientific research was high (*M* = 3.73, *SE* = 0.040), with a high intention to use AI‐based tools (*M* = 3.49, *SE* = 0.051); the highest mean indicated the highest benefits and intention to use, and both were above the mean of 3/5 (Table 2). Scores above the midpoint of 3 suggest generally favorable attitudes among participants [20].

**Table 2:** Means, standard errors of the means, maximum, minimum, and 95% confidence interval (CI) of the means of using AI in nursing research (*N* = 311).

**Items**	**Reliability coefficient**	**Mean**	**SE of the mean**	**Mini.**	**Max.**	**95% confidence interval**	
**Scale**	**0.911**					**Lower**	**Upper**

Perceived performance anxiety	0.896	3.46	0.047	1.20	5.00	3.37	3.56
I am concerned that the mechanisms used by AI‐based devices may lead to inaccurate predictions.		3.44	0.053	1	5	3.34	3.54
I am concerned that the mechanisms used by AI‐based devices may result in research errors.		3.56	0.063	1	5	3.44	3.68
I am concerned that treatments or suggestions provided by AI devices may be incomplete.		3.47	0.058	1	5	3.36	3.58
I am concerned that the predictive models of AI‐based tools may malfunction.		3.34	0.055	1	5	3.23	3.45
I am concerned that the research decisions made by AI devices may be inadequate.		3.53	0.053	2	5	3.42	3.63
Perceived social biases	0.870	3.21	0.048	1.60	5.00	3.11	3.30
I am concerned that AI‐based devices may overestimate or underestimate risks in certain data.		3.43	0.052	2	5	3.33	3.54
I am concerned that data used in AI devices may lead to discrimination against a certain patient group (e.g., minority groups).		2.91	0.061	1	5	2.79	3.03
I am concerned that AI‐based tools used in research may be unfair to a certain group of the population.		3.08	0.064	1	5	2.95	3.21
I am concerned that AI devices could lead to morally flawed practices in research.		3.52	0.059	1	5	3.41	3.64
Overall, I am concerned that the possibility of biases by AI devices against certain groups of the population is high.		3.13	0.060	1	5	3.01	3.25
Perceived privacy concerns	0.927	3.47	0.044	2.00	5.00	3.38	3.56
I think using AI‐based applications helps research entities collect too much personal information from people.		3.41	0.057	1	5	3.30	3.52
I think, in this case, I am concerned that research entities use clients’ information for other purposes without their knowledge and authorization.		3.41	0.052	2	5	3.31	3.52
In this case, I am concerned that clients’ information will be shared with other entities without their explicit consent.		3.49	0.050	2	5	3.39	3.58
In this case, I am concerned that unauthorized people will have access to clients’ information.		3.57	0.051	2	5	3.47	3.67
In this case, I am concerned about the privacy of clients’ information during AI‐based research practices.		3.47	0.049	2	5	3.38	3.57
In this case, I am concerned that clients’ information would be sold to others without their permission.		3.52	0.054	2	5	3.41	3.62
Perceived mistrust in AI mechanisms	0.909	30.02	0.044	1.00	4.40	2.93	3.11
I trust in the AI‐scientific research.		2.94	0.057	1	5	2.82	30.05
I trust in the AI algorithms used in scientific research.		3.21	0.053	1	4	3.10	3.31
I trust in AI’s predictive and diagnostic ability.		3.10	0.047	1	5	3.01	3.19
I trust in the accuracy and predictive powers of current AI algorithmic models used in scientific research.		2.95	0.055	1	5	2.84	3.06
I trust that AI‐based tools can adapt to specific and unforeseen scientific research situations.		2.93	0.050	1	4	2.83	3.03
Perceived communication barriers	0.876	3.74	0.036	1.80	5.00	3.67	3.81
I am concerned that AI tools may eliminate the contact between researchers and clients.		3.51	0.049	2	5	3.41	3.60
I am concerned that AI tools may reduce conversations between researchers and clients.		3.68	0.044	2	5	3.60	3.77
I am concerned that AI devices may decrease the human aspects of relations in the research context.		3.85	0.040	2	5	3.77	3.93
I am concerned that by using AI devices, I may lose face‐to‐face cues and personal interactions with researchers.		3.94	0.044	1	5	3.85	4.03
I am concerned that by using AI devices, I may be in a more passive position in making research decisions.		3.73	0.043	2	5	3.65	3.81
Perceived unregulated standards	0.877	3.76	0.035	2.40	5.00	3.69	3.83
I am concerned that special policies and guidelines for AI tools are not yet transparent.		3.87	0.041	2	5	3.79	3.95
I am concerned that the safety and efficacy of AI tools are not clearly regulated.		3.69	0.049	2	5	3.59	3.78
I am concerned that regulatory standards to assess AI algorithmic safety are yet to be formalized.		3.78	0.044	2	5	3.69	3.86
I am concerned that an appropriate regulatory and accreditation system regarding AI‐based devices is not in place yet.		3.62	0.044	2	5	3.54	3.71
I am concerned about the lack of clear guidelines to monitor the performance of AI tools in the research context.		3.85	0.038	2	5	3.77	3.92
Perceived liability issues	0.912	3.78	0.036	2.33	5.00	3.71	3.85
I am concerned because it is not clear who is responsible when errors result from the use of AI research tools.		3.71	0.045	2	5	3.62	3.80
I am concerned about the liability of using AI‐based services for my scientific research.		3.65	0.038	2	5	3.58	3.73
I am concerned because it is not clear who becomes responsible if AI‐based tools offer wrong recommendations.		3.89	0.044	2	5	3.80	3.98
I am concerned because it is unclear where the lines of responsibility begin or end when AI devices guide scientific research.		3.88	0.045	2	5	3.79	3.97
I am concerned because it is not clear who is responsible if appropriate AI recommendations are mistakenly dismissed.		3.94	0.047	2	5	3.85	4.03
Overall, I am concerned that the use of AI tools for scientific research purposes increases my liability.		3.62	0.042	2	5	3.54	3.71

	**Very High**						

Perceived risks (very low/very high)	0.845	1.64	0.021	1.00	2.00	1.60	1.68
The risk of using AI‐based tools for research purposes is………		1.69	0.026	1	2	1.64	
The degree of uncertainty associated with the use of AI research tools is………		1.69	0.026	1	2	1.64	
The potential loss associated with the use of AI devices is………		1.57	0.028	1	2	1.52	
The likelihood of unexpected problems with the use of AI devices is………		1.57	0.028	1	2	1.52	
Overall, the chance of adverse consequences associated with the use of AI‐based tools for research purposes is………		1.69	0.026	1	2	1.64	
Perceived benefits	0.931	3.73	0.040	1.86	5.00	3.65	3.81
I believe AI‐based scientific research services can improve diagnostics.		3.69	0.050	1	5	3.59	3.79
I think AI‐based scientific research devices can enhance prognosis.		3.66	0.045	2	5	3.57	3.75
I believe AI‐based scientific research devices can advance data management systems.		3.85	0.043	2	5	3.77	3.94
I believe AI‐based scientific research tools can suggest accurate research planning.		3.71	0.052	2	5	3.61	3.81
I think AI‐based scientific research services can recommend reliable options.		3.54	0.052	2	5	3.44	3.64
I think AI‐based scientific research tools can reduce costs.		3.88	0.045	2	5	3.80	3.97
Overall, I think AI‐based scientific research devices can boost research outcomes.		3.81	0.045	2	5	3.73	3.90
Perceived intention to use AI‐based tools	0.966	3.49	0.051	1.00	5.00	3.39	3.59
I agree to use AI‐based tools for research purposes.		3.42	0.056	1	5	3.31	3.53
Using AI‐based tools for scientific research purposes is something I would consider.		3.60	0.055	1	5	3.49	3.71
I want to use AI‐based devices to manage my scientific research.		3.54	0.052	1	5	3.44	3.64
In the future, I am willing to use AI‐based services for scientific research.		3.54	0.055	1	5	3.43	3.65
I am very likely to use recommendations provided by AI‐based tools for scientific research.		3.38	0.057	1	5	3.27	3.49
Total score of the scale.		181.48	1.10	121.00	225.00	179.31	183.65
Total mean score of the scale.		3.36	0.020	2.24	4.17	3.32	3.40
How positive or negative do they feel about the use of artificial intelligence in nursing research?		3.38	0.058	1	5	3.27	3.50

*Note:* The Likert scale is rated from 1 (strongly disagree) to 5 (strongly agree). SE = standard error of the mean; 95% confidence interval (CI) of the mean, using standard errors.

### 4.3. Perceived Risks (Very Low/Very High)

This scale was on a two‐point one, and it was a question rather than a subscale. The higher the mean score, the higher the perceived risk of using AI in nursing research. Of the perceived risks of using AI in scientific research, the highest reported mean was related to the degree of uncertainty and the chance of adverse consequences associated with the use of AI research tools (*M* = 1.69, *SE = *0.026; for both), while the lowest perceived mean was related to the potential loss and unexpected problems associated with the use of AI devices (*M* = 1.57, *SE = *0.028; for both) (Table 2).

#### 4.3.1. Perceived Performance Anxiety

Respondents most frequently expressed concern that the mechanisms used by AI‐based devices could result in research errors. The least concern was reported regarding the possibility that predictive models of AI‐based tools might malfunction (see Table 2).

#### 4.3.2. Perceived Social Biases

The most prominent concern was that AI devices could lead to morally flawed practices in research. In contrast, the least concern was about the potential for AI devices to cause discrimination against certain patient groups, such as minorities (see Table 2).

#### 4.3.3. Perceived Privacy Concerns

The greatest concern was related to unauthorized access to clients’ information. Conversely, the lowest concerns were about AI‐based applications enabling research entities to collect excessive personal information and the use of clients’ information for other purposes without their knowledge or authorization (see Table 2).

#### 4.3.4. Perceived Mistrust in AI Mechanisms

Nursing educators reported the highest level of trust in the AI algorithms used in scientific research. However, there was less trust that AI‐based tools could adapt to specific and unforeseen scientific research situations (see Table 2).

#### 4.3.5. Perceived Communication Barriers

The main concern was that using AI devices might lead to a loss of face‐to‐face cues and personal interactions with researchers. The lowest concern was that AI tools may eliminate contact between researchers and clients (see Table 2).

#### 4.3.6. Perceived Unregulated Standards

The highest concern was about the lack of transparency in policies and guidelines for AI tools. In contrast, the lowest concern was related to the absence of appropriate regulatory and accreditation systems for AI‐based devices (see Table 2).

#### 4.3.7. Perceived Liability Issues

Nursing educators were most concerned about the lack of clarity regarding responsibility if appropriate AI recommendations were mistakenly dismissed. The lowest concern was about the increased liability for academics using AI tools for scientific research purposes (see Table 2).

**Figure 1:**
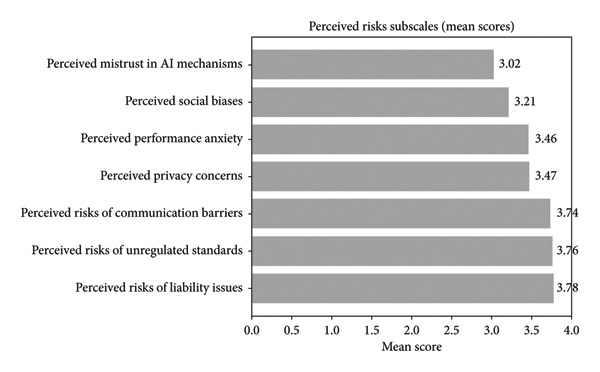
Mean scores for perceived risk subscales.

The overall hazard mean is “high,” although some subscales (e.g., social biases and mistrust in AI mechanisms) are closer to neutral, indicating variation in perceived risk across domains. Figure 1 illustrates that certain subscales, such as social biases and mistrust in AI mechanisms, have mean scores closer to neutral. This highlights that perceived risk is not uniform across all domains, and interpretations should reflect these differences rather than generalize the overall mean as “high” for all subscales.

### 4.4. Perceived Benefits

Higher mean scores indicate greater perceived benefits of AI in nursing research. The most notable benefits reported by nursing educators were that AI‐based scientific research tools could reduce costs (*M* = 3.88, SE = 0.045) and enhance research outcomes (*M* = 3.81, SE = 0.045). In contrast, the lowest perceived benefit was that AI‐based research services could recommend reliable options (*M* = 3.54, SE = 0.052) (Table 2).

### 4.5. Perceived Intention to Use AI‐Based Tools

Perceptions of risks and benefits influenced nursing educators’ intentions to use AI tools. The highest reported intention was for using AI‐based tools in scientific research (*M* = 3.60, SE = 0.055), while the lowest was for being very likely to follow AI‐generated recommendations (*M* = 3.38, SE = 0.057) (Table 2).

A final overall question assessed educators’ general attitudes toward AI in nursing research: “Taken all together, how positive or negative do you feel about the use of AI in nursing research?” With scores above the midpoint of 3, results indicated generally positive attitudes (*M* = 3.38, SE = 0.058, 95% CI = 3.27–3.50) (Table 2).

### 4.6. Predictors of the Perceived Use of AI in Nursing Research

To address the second research question, a GLM analysis identified marital status (*B* = 7.67, *p* = 0.001), age (*B* = −14.65, *p* = 0.001), education level (*B* = 13.80, *p* = 0.001), academic rank (*B* = −1.755, *p* = 0.001), and teaching experience (*B* = 2.793, *p* = 0.001) as significant predictors of AI use. The model was significant (*F* (df = 7) = 86.82, *p* = 0.001, *R* ^2^ = 0.660), explaining 66% of the variance in perceived AI use. Married educators with postgraduate degrees and more teaching experience were more likely to use AI. In contrast, older educators and those in technical academic roles (such as clinical trainers) tended to use AI less (Table 3).

**Table 3:** Generalized linear model (GLM) of nursing educators’ characteristics as predictors of using AI in nursing research (*N* = 311).

Dependent and significant predictors	*B* ^∗^	*T*‐test	*p*	*R* ^2^	Adjusted *R* ^2^	*F*‐test (df) ^∗∗^(*p*)
The total score of using AI in nursing research^∗∗∗^				0.667	0.660	86.82 (7) (0.001)
Marital status—married	7.67	8.10	0.001			
Age‐ 35–44 years	−14.65	−14.32	0.001			
Level of education—postgraduate	13.80	11.81	0.001			
Academic rank—clinical trainers	−1.75	−4.17	0.001			
Teaching experience—5–9 years	2.79	3.77	0.001			

*Note:* *B* ^∗^ = Unstandardized coefficients. Covariates appearing in the model are evaluated at the following values: gender = 1.62, marital status = 1.90, age = 3.27, level of education = 1.49, type of university educators teaching in = 1.00, type of university educators graduated from = 1.00, academic rank = 4.30, years of academic experience = 3.72.

Abbreviation: df = degree of freedom.

^∗∗∗^Total score of using AI in nursing research: mean (standard error) (confidence interval (CI), upper bound–lower bound) = 181 (1.10), 95% CI (180.21–182.74).

^∗∗^ *p* < 0.001 (2‐tailed).

### 4.7. Differences in the Perceived Use of AI in Nursing Research

ANOVA results showed significant differences in AI use based on age (*F* = 27.63, df = 4, *p* = 0.001), academic rank (*F* = 60.79, df = 5, *p* = 0.001), and teaching experience (*F* = 17.02, df = 4, *p* = 0.001). Younger and midcareer educators, as well as associate professors, were more likely to use AI as they progressed in their academic careers (Table 4).

**Table 4:** Differences in the total mean score of using AI in nursing research based on nursing educators’ characteristics (*N* = 311).

Nursing educators’ characteristics	*N*	Mean	SD	*F*‐test (df)	*p*	Scheffe’s post hoc test
Age				27.63 (4)	0.001	Group 1 > 5 > 2
1. Less than 25 years	9	3.46	0.01			
2. 25–34 years	34	3.72	0.18			
3. 35–44 years	169	3.33	0.24			
4. 45–54 years	62	3.45	0.33			
5. 55 or more	37	2.97	0.57			
Academic rank				60.79 (5)	0.001	Group 1 > 2 > 4 > 5 > 6
1. Full professor	44	3.62	0.35			
2. Associate professor	27	2.65	0.40			
3. Assistant professor	64	3.54	0.21			
4. Teacher	33	3.38	0.11			
5. Clinical trainer	87	3.39	0.16			
6. Laboratory technician	56	3.21	0.29			
Teaching experience				170.02 (4)	0.001	Group 5 > 1 > 3
1. Less than 1 year	15	2.75	0.01			
2. 1–2 years	51	3.43	0.14			
3. 3–4	61	3.50	0.26			
4. 5–9	64	3.40	0.10			
5. Ten or more	120	3.31	0.47			

Abbreviations: df = degree of freedom, SD = standard deviation.

**Figure 2:**
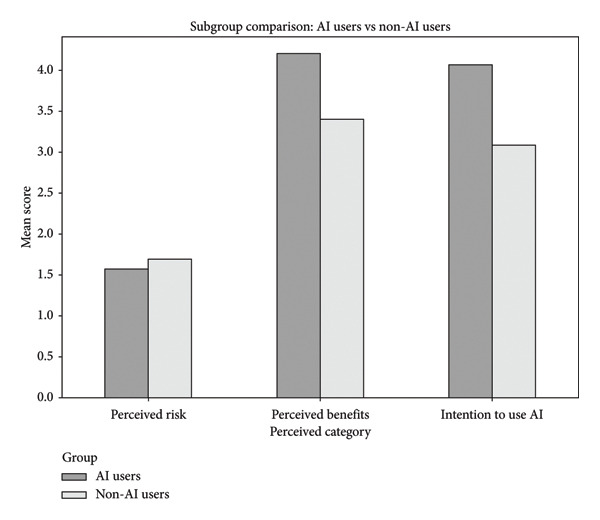
Subgroup comparison of perceived risks, benefits, and intention to use AI.

To enhance the validity of the conclusions, subgroup analysis was conducted comparing nursing educators who reported using AI tools in research with those who did not. AI users reported lower perceived risks (Mean = 1.568 ± SD = 0.435) and higher perceived benefits (4.198 ± 0.512) and intention to use AI (4.071 ± 0.701) compared to non‐AI users (1.699 ± 0.312, 3.403 ± 0.636, 3.083 ± 0.812) (Figure 2). These findings suggest that direct experience with AI may positively influence attitudes and readiness for adoption, reinforcing the importance of exposure and training in shaping perceptions.

## 5. Discussion

The current study focused on perceptions rather than tangible outcomes; thus, the results should be interpreted with caution. In the current study, there was a notable hesitance among older educators, those above 44 years old, and those in clinical roles. The current results contradict the wealth of research highlighting the growing significance of integrating AI into nursing research. For example, El Arab et al. [23] pointed out how AI is fundamentally reshaping nursing by streamlining clinical workflows and enhancing decision‐making. The researchers urged for a more strategic approach to the incorporation of AI technologies. These advancements imply that nurturing a culture of innovation in academic institutions might be essential to stay in sync with the evolving global trends in nursing research.

In this study, the TAM provided a valuable framework for interpreting nursing educators’ perceptions of AI in research. Age, academic rank, and teaching experience were significant predictors of these perceptions and intentions to use AI. Younger and mid‐career educators were more open to adopting AI, likely due to perceiving it as useful and easy to use. At the same time, those in clinical roles or with more teaching experience were more cautious, possibly due to concerns about usability, reliability, or ethics. These findings align with TAM’s premise that demographic and experiential factors shape attitudes toward new technologies. Although perceived usefulness and ease of use were not directly measured, the identified predictors suggest these beliefs vary among different groups of nursing educators. These findings also align with those of Algunmeeyn and Mrayyan [8], who reported that nursing students perceived AI as a valuable tool in research but expressed concerns about ethical implications and data reliability. The convergence of student and nurse perspectives underscores the importance of targeted education and policy frameworks to support responsible AI adoption in nursing.

The final question posed by the first researcher about the overall perception toward using AI in nursing research effectively captures this sentiment by allowing participants to express their feelings regarding AI. The results suggest that nursing educators lean toward a favorable view of AI in this context. The standard error of 0.058 and the CI ranging from 3.27 to 3.50 (5 = very positive, 4 = positive, 3 = neutral, 2 = negative, and 1 = very negative) indicate a relatively precise estimate of the mean, reinforcing the reliability of these findings. This positive perception may stem from the potential benefits of AI in enhancing research efficiency, improving data analysis, and facilitating evidence‐based practice within nursing [24, 25]. This result is supported in the literature; that is, a positive attitude toward AI could lead to increased integration of technology in nursing education and research methodologies [26]. It also opens avenues for further research into specific applications of AI that educators find most beneficial, as well as potential barriers to its implementation [27].

The findings indicate a high overall mean for perceived risks associated with the use of AI in scientific research, underscoring significant concerns within the academic community, similar to the international trend [28, 29]. The high mean score for perceived risks suggests that, despite the advantages AI may offer in enhancing research efficiency and data analysis, these benefits are often overshadowed by concerns regarding reliability and ethical integrity. This sentiment aligns with the findings of Chen et al. [30], who argued that addressing these perceived risks is crucial for fostering a more positive perception of AI in research settings.

On the other hand, the high overall mean for perceived benefits of using AI in scientific research, coupled with a strong intention to utilize AI‐based tools, reflects a growing recognition of the transformative potential of AI technologies in enhancing research methodologies and outcomes. This result is evident in other studies that highlighted various advantages associated with AI, such as improved efficiency, enhanced data analysis capabilities, and the ability to uncover novel insights from complex datasets [31]. Their research demonstrated that the integration of AI not only accelerates the research process but also improves the accuracy of data interpretation, leading to more reliable outcomes. Additionally, Bisconti et al. [32] emphasized that AI can facilitate interdisciplinary collaboration by providing tools that can tackle complex problems and synthesize information across various domains using nontraditional analytical methods. Furthermore, the high intention to use AI‐based tools suggests that researchers are not only aware of these benefits but are also motivated to integrate AI into their workflows. This result aligns with the insights from Krakowski et al. [33], who found that researchers perceive AI as a means to enhance their competitive edge in an increasingly data‐driven research landscape.

The influence of nursing educators’ age, academic rank, and teaching experience on the use of AI in nursing research highlights the multifaceted factors that impact technology adoption in academic settings. Our study showed that there was a notable hesitance among older educators (age) and those in clinical roles (or rank) to use AI in nursing research, which was supported by other studies. For example, Alruwaili et al. [34] found that younger educators tend to be more comfortable with technology and are more likely to embrace AI tools in their research and practice generally; they tend to be open to innovation, suggesting that younger faculty members are not only more familiar with digital technologies but also more inclined to experiment with new methodologies. However, opposite to our finding related to academic rank, Chaaban et al. [35], senior faculty members, such as full‐ranked professors, often possess established research methodologies. They may be more resistant to changing their approaches. Their research highlights that while higher ranked educators may recognize the benefits of AI, they are less likely to implement it due to concerns about the reliability of new technologies and the potential disruption of established workflows. The current educators who have clinical roles (rank) were more hesitant to use AI in their research because they do not need to conduct research to be promoted; they have specific rules and regulations to be promoted in our universities. In our study, teaching experience also emerged as a significant predictor of AI use in nursing research. As noted by Weinberg [36], educators with extensive teaching backgrounds often have a wealth of knowledge about traditional research methods, which can create a hesitance to adopt AI.

### 5.1. Perceived Risks of Using AI in Nursing Research

The perceived risks associated with the use of AI in nursing research, as indicated by nursing educators, converge on the following interconnected themes:

• *Theme 1: Liability and Accountability:* Ultimately, concerns regarding liability and accountability remain prevalent. Educators express particular discomfort with the ambiguous lines of responsibility when AI‐generated recommendations are disregarded or result in negative outcomes. There is a demand for strong frameworks that delineate accountability and promote a culture of trust and ethical conduct [37–39].
• *Theme 2: Regulatory and Policy Gaps:* The absence of clear policies and regulatory standards for the use of AI in research is a recurring issue. Educators advocate for straightforward, accessible guidelines to ensure the ethical and consistent implementation of AI technologies. While there is some confidence that existing frameworks can adapt, there is a significant push for proactive policy development that addresses the specific challenges posed by AI in nursing research [29, 40, 41].
• *Theme 3: Communication and Human Interaction:* The potential decline of interpersonal communication is another significant concern. Educators appreciate the relational dimensions of research and fear that an increased dependence on AI may reduce face‐to‐face collaboration and the nuanced understanding that arises from human interaction. Nevertheless, many also recognize the potential for AI to optimize administrative tasks, thereby allowing more time for meaningful engagement [42–44].
• *Theme 4: Trust and Adaptability of AI Mechanisms:* The concept of trust in AI is complex. Although educators typically have confidence in AI algorithms for data analysis, they exhibit less assurance regarding AI’s capacity to adjust to specific or unexpected research situations. This theme underscores the necessity for continuous education concerning both the strengths and weaknesses of AI tools, alongside the need for the creation of more adaptable, context‐sensitive AI systems [15, 26, 45, 46].
• *Theme 5: Data Privacy and Security:* Concerns regarding data privacy represent another significant issue. Educators are especially cautious about unauthorized access to sensitive data and the wider implications for confidentiality and trust. While some assert that current ethical guidelines provide adequate protection, the swift evolution of AI demands persistent vigilance and the adjustment of privacy standards [47–49].
• *Theme 6: Ethical and Social Implications:* The ethical aspect is another key theme, particularly in relation to the potential for AI to reinforce social biases or result in morally ambiguous practices. Educators acknowledge that certain safeguards can alleviate some risks; however, there remains a lingering concern regarding the ethical ramifications of entrusting critical research decisions to AI systems, particularly in sensitive fields such as healthcare [45, 50, 51].
• *Theme 7: Uncertainty and Reliability:* A primary concern is the uncertainty associated with AI’s reliability and predictability. Educators have voiced worries that the opaque characteristics of AI algorithms and their decision‐making processes may introduce unpredictability into research results. This uncertainty encompasses both technical and ethical dimensions, as it raises issues regarding the integrity and reproducibility of AI‐assisted research [52–54].

In conclusion, the perceived risks associated with AI in nursing research are complex yet interconnected, focusing on aspects, such as uncertainty, ethics, privacy, trust, communication, regulation, and liability. A comprehensive approach to these issues, through specialized training, clear policies, and continuous dialog, will be crucial for the ethical integration of AI into nursing research. These risks are not standalone issues; they represent a wider anxiety regarding the incorporation of intricate, swiftly advancing technologies into research settings where accuracy, transparency, and ethical principles are of utmost importance.

### 5.2. Perceived Benefits of Using AI in Nursing Research

Our nursing educators were generally optimistic about the potential benefits of these technologies, particularly in reducing costs and enhancing research productivity. The high mean scores reflecting beliefs that AI tools could lower expenses and boost research output suggest that educators recognize the transformative potential of AI in streamlining research processes. Like other research, it was commonly documented that AI integration in research resulted in cost‐saving benefits [55]. The researchers found that AI technologies automate time‐consuming tasks such as data analysis, allowing researchers to allocate resources more efficiently. Educators appreciate the potential for AI to minimize operational costs, thereby freeing up funding for other critical areas, such as training and development [55].

Moreover, nursing educators’ belief that AI could enhance research productivity aligns with findings from Sallam [56], who noted that AI tools can facilitate faster data processing and more accurate results, ultimately leading to more effective research outcomes. This perspective underscores the positive impact that AI can have on the overall research landscape, particularly in nursing, where timely and accurate data are crucial for evidence‐based practice.

In contrast, the lower perceived mean regarding the belief that AI‐based scientific research services could recommend reliable options indicates some skepticism about the dependability of AI recommendations. As noted by Aldoseri et al. [57], while AI analyzes large amounts of data and identifies trends, there remain concerns about the reliability of its recommendations, particularly in complex research environments. Nursing educators may be wary of relying too heavily on AI for critical decision‐making, fearing that it could lead to inappropriate or suboptimal outcomes. This discrepancy in perceptions highlights the need for further validation and transparency in AI algorithms used in research. As Benda et al. [58] suggested, ensuring that AI systems provide reliable and contextually appropriate recommendations is crucial for fostering trust among nursing educators. Ongoing training and education about the capabilities and limitations of AI tools will also be essential in addressing these concerns.

### 5.3. Perceived Intention to Use AI‐Based Tools

Nursing educators reported a nuanced view regarding their intentions to use AI‐based tools. This result balances optimism about technology’s potential with caution about its application. The high mean score reflecting a willingness to consider using AI‐based tools suggests that educators recognize the transformative possibilities these technologies can offer in terms of improving research efficiency and effectiveness.

There are many advantages to using AI in educational and research contexts. For instance, Lau et al. [59] found that educators perceive AI tools as beneficial for automating routine tasks, such as data collection and preliminary analysis, which can free up time for more critical aspects of research and enhance overall productivity. This positive outlook is indicative of a broader trend in academia, where educators seek innovative solutions to cope with increasing demands as well as complexities in research methodologies.

Conversely, the lower perceived mean regarding the likelihood of using AI‐generated recommendations indicates a significant level of skepticism among nursing educators about fully trusting AI in decision‐making. According to Chin‐Yee and Upshur [60], many health practitioners express concerns about the reliability and contextual relevance of AI recommendations, fearing that these insights might not align with the specific nuances of clinical practice or research needs. This wariness reflects a desire to maintain a level of human oversight in research processes, particularly when it comes to interpreting data and making critical decisions. This discrepancy highlights the importance of addressing both the potential benefits and the concerns accompanying AI in nursing research. As Borger et al. [61] note, building trust in AI technologies requires not only demonstrating their efficacy through validation but also ensuring that educators are equipped with the knowledge to assess AI‐generated recommendations critically. Ongoing professional development and training can help bridge the gap between the enthusiasm for AI tools and the hesitation to rely on their outputs.

### 5.4. Predictors of the Perceived Use of AI in Nursing Research

A distinctive contribution of this study is its emphasis on the viewpoints of educators and the demographic factors influencing AI adoption in nursing research. The factors influencing nursing educators’ utilization of AI in nursing research, including marital status, age, educational level, academic rank, and teaching experience, underscore the complex elements related to technology adoption in academic contexts. The model’s capacity to account for 66% of the variance in perceived AI usage highlights the crucial influence these demographic and professional traits have on shaping attitudes toward AI integration in nursing research. By investigating how these factors relate to AI utilization, this research offers important insights into the distinct trends of technology adoption among nursing educators. These results aid in elucidating the intricate relationship between personal and professional traits and the incorporation of AI in academic research, providing a detailed understanding that can guide targeted interventions and policy formulation.

Supporting some of our findings, research has shown that married individuals frequently gain advantages from shared resources and support systems, which can improve their capacity to engage with new technologies [62]. The existence of a supportive partner may enhance the time and resources available for professional growth and the exploration of innovative research methodologies, including AI [63]. Furthermore, nursing educators with advanced degrees generally have a more profound comprehension of sophisticated research methodologies and tend to be more acquainted with the latest technological advancements. This outcome is consistent with the observations made by Han et al., who indicated that higher educational qualifications are linked to a greater willingness to adopt AI tools in research environments [64]. In addition, extensive teaching experience can equip educators with a substantial amount of practical knowledge, allowing them to effectively incorporate AI into their research endeavors [65].

On the other hand, the decline in AI usage among older nursing educators is particularly noteworthy. With age often comes established practices and methodologies that may foster resistance to the adoption of new technologies [66]. Older educators might lack confidence in their ability to effectively utilize AI tools, which can lead to hesitance in embracing these innovations in their research. Additionally, educators occupying more technical academic positions, such as clinical trainers, may place a higher value on direct student engagement rather than research endeavors. This emphasis on clinical education over research can restrict their familiarity with AI tools, as they may not perceive the immediate significance of these technologies in their daily responsibilities [67, 68]. Their primary function often revolves around practical training and mentorship of students [8], which may not require the use of AI, thereby contributing to lower adoption rates in research settings.

In the present study, the identified relationships between marital status, educational attainment, teaching experience, and AI usage in nursing research suggest a nuanced landscape of factors influencing technology adoption in this field.

### 5.5. Differences in the Perceived Use of AI in Nursing Research

The results concerning significant differences in nursing educators’ use of AI in nursing research based on age, academic rank, and teaching experience illustrate the varying levels of engagement with technology across different demographics. Young and mid‐career nursing educators, particularly those in associate professor roles, were more inclined to adopt AI tools as they progress toward higher academic positions, such as full professorships. To increase the validity of the current study’s conclusions, subgroup analysis was conducted comparing nursing educators who reported using AI tools in research with those who did not. Direct experience with AI may positively influence attitudes for adoption, shedding light on the importance of exposure to AI and training in shaping nursing educators’ perceptions.

Supporting some of our findings, younger nursing educators typically exhibit greater openness to new technologies, including AI. Research by Alruwaili et al. [34] supports this observation, showing that younger educators are often more familiar with digital tools and innovative research methodologies. This familiarity can lead to a more proactive approach to integrating AI into their research practices, allowing them to leverage these technologies to enhance their work and academic profiles. Academic rank was correlated with the adoption of AI technologies. Associate professors, who are often in the transitional stages of their careers, may feel a pressing need to enhance their research output and visibility, leading them to embrace AI tools more readily. As noted by Alruwaili et al. [34], educators at this level are often motivated to publish more frequently and seek funding opportunities, which can drive them to utilize AI for data analysis and literature review processes.

On the other hand, nursing educators who have higher academic ranks may have established research methodologies that they are less inclined to alter. According to Chaaban et al. [35], senior faculty members are expected to resist adopting new technologies due to a combination of comfort with existing practices and a lack of perceived necessity. This inertia can hinder the integration of AI into their research endeavors. Teaching experience also influences the use of AI in research. Young and mid‐career educators tend to be more engaged with ongoing professional development, which often includes training on the latest technologies [69]. Such engagement has been shown to foster a culture of innovation and experimentation with AI tools, thereby enabling these educators to incorporate technology into their research effectively. In contrast, more experienced educators may prioritize traditional teaching methods and may not feel the same urgency to adopt AI, particularly if they are less familiar with its applications [70].

### 5.6. Limitations of the Study

Although this research contributes meaningfully to the growing body of knowledge on the use of AI in nursing research, several limitations must be acknowledged. First, the study employed a cross‐sectional design and nonrandom convenience sampling, which restricts the generalizability of the findings. Data were collected from a limited number of governmental and private universities, and while this provided initial insights, it does not allow for broad application across diverse educational contexts. Future research should consider longitudinal designs and random cluster sampling to enhance representativeness and track changes over time. Second, the diversity of nationalities among participants introduced variability that, while enriching the dataset, may have complicated interpretation. A more focused sample, such as limiting participants to nursing educators from Jordan, could have yielded a more contextually grounded and internally valid understanding of AI perceptions within a specific academic and cultural framework. Third, socioeconomic factors may influence the adoption and perception of AI in nursing research. These contextual differences limit the applicability of the findings to other countries or regions with different technological infrastructures, educational systems, or healthcare priorities.

Fourth, the study relied on self‐selection through social media recruitment, which may have introduced selection bias. Participants who chose to respond may have had a preexisting interest in AI or digital technologies, potentially skewing the results toward more favorable perceptions and higher familiarity. Fifth, the survey was administered exclusively in English, which may have posed a barrier for non‐native speakers, which could have affected comprehension and the accuracy of responses, especially in nuanced questions about AI concepts. Future studies should consider offering multilingual survey options to ensure inclusivity and clarity. Finally, some participants may have limited prior exposure to AI, making it difficult to interpret their responses accurately. Without a shared baseline understanding of AI, responses may reflect confusion or misinterpretation rather than genuine attitudes or knowledge. Including a brief educational primer or screening for AI familiarity could mitigate this issue in future research.

### 5.7. Implications for Future Research

In the realm of nursing research, there is a significant opportunity to encourage the use of AI tools to enhance data analysis and improve research efficiency. Researchers should actively promote the adoption of AI technologies, which can lead to more robust findings and ultimately support evidence‐based practice. Addressing skepticism surrounding AI outputs is crucial; conducting studies that validate the reliability and effectiveness of AI recommendations can help alleviate concerns among nursing educators and practitioners. Furthermore, research initiatives should emphasize the importance of training nursing educators and researchers on AI applications to bridge the gaps between technology and practical use in nursing research.

By addressing these implications across various domains of nursing, stakeholders can foster a more effective integration of AI technologies, ultimately enhancing the quality of care, education, administration, and research within the nursing profession.

### 5.8. Implications for Nursing Educators and Policymakers

Starting by establishing a collaborative learning environment and enhancing research skills [16] and AI skills, nursing leaders must allocate resources effectively to acquire and implement AI technologies within healthcare settings. These resources include ensuring that staff have full access to the necessary tools and training to utilize AI effectively. In line with the study’s findings, it is recommended that nursing administrators develop clear and detailed policies to address liability concerns associated with AI use. These policies should explicitly outline teacher and staff responsibilities, data security protocols, and procedures for reporting incidents, thereby reducing uncertainty and promoting accountability. It is also imperative for nursing administrators to develop clear policies and ethical standards related to the use of AI, which should include guidelines for data security, ethical use, and accountability.

Furthermore, specific training programs should be developed for older or less technologically confident educators, focusing on hands‐on, step‐by‐step instruction and ongoing support. This targeted approach will help bridge the confidence gap and ensure all staff can effectively integrate AI into their practice. Algunmeeyn and Mrayyan [8] reported that integrating AI literacy into nursing is critical. A culture of innovation and providing support for staff interested in exploring AI applications should be enforced by the administrators; this could facilitate a smoother integration of technology into everyday nursing practice. Policymakers ought to focus on establishing national frameworks that guarantee fair access to AI tools, protect data privacy, and encourage interdisciplinary collaboration in nursing research.

## 6. Conclusions

The integration of AI in nursing research presents both risks and benefits that are influenced by a variety of factors, including educators’ age, academic rank, and teaching experience. While younger and mid‐career nursing educators show a propensity to embrace AI tools, older educators and those in clinical roles may exhibit reservations. Although the study did not directly assess the impact of structured training programs, the findings suggest that demographic factors may influence educators’ readiness and attitudes toward AI. This case implies that targeted educational initiatives and institutional support could enhance AI adoption in nursing research, particularly among hesitant groups.

Beyond individual readiness, this study contributes to the broader literature by contextualizing AI acceptance within nursing education and research, highlighting the importance of aligning technological integration with ethical standards and professional values. While this study offers valuable insights, its cross‐sectional design, nonrandom sampling, and contextual limitations should be considered when interpreting the results. Thus, future research should delve deeper into specific AI applications, including writing tools and predictive modeling, to gain a better understanding of their adoption, effects, and sustainability in nursing research.

To ensure the responsible and effective integration of AI into nursing research, it is essential to equip nursing educators with the necessary tools, training, and critical thinking skills to engage with AI technologies effectively. Empowering educators in this way will foster innovation and improve research quality and uphold the ethical standards and professional values central to nursing research, education, and practice. Policymakers should prioritize the development of national frameworks that ensure equitable access to AI tools, safeguard data privacy, and promote interdisciplinary collaboration in nursing research.

## Ethics Statement

The study has an IRB (22/4/2022/2023, dated January 15^th^, 2023) of the Hashemite University, Jordan.

## Consent

The invitation letter informed the participants that their participation in the survey constituted their consent to participate in the study.

## Conflicts of Interest

The authors declare no conflicts of interest.

## Author Contributions

Majd T. Mrayyan: conceptualization, abstract, methodology, investigation, data analysis and writing, limitations, original draft preparation, writing, review, editing, and supervision. Ahmad K. Al‐Omari: writing, review, and editing of discussion and implications. The first and second authors have equal contributions; thus, they can be considered first authors.

## Funding

This research received no specific grant from any funding agency in the public, commercial, or not‐for‐profit sectors.

## Supporting information


**Supporting Information** Additional supporting information can be found online in the Supporting Information section.

## Data Availability

The datasets used and/or analyzed during the current study are available from the corresponding author upon reasonable request.
